# Evaluation of Extrusion Technique for Nanosizing Liposomes

**DOI:** 10.3390/pharmaceutics8040036

**Published:** 2016-12-21

**Authors:** Sandy Gim Ming Ong, Mallikarjun Chitneni, Kah Seng Lee, Long Chiau Ming, Kah Hay Yuen

**Affiliations:** 1 School of Pharmaceutical Sciences, University of Science Malaysia, 11800 Penang, Malaysia; khyuen@usm.my; 2 Jurox Pty Ltd., Rutherford 2320, NSW, Australia; Mallik.Chitneni@jurox.com.au; 3 Pharmacy, School of Medicine, University of Tasmania, Hobart 7001, Tasmania, Australia; kah_seng_81@yahoo.com (K.S.L.); long.ming@utas.edu.au (L.C.M.)

**Keywords:** extrusion, pro-liposomes, liposomes, particle size, membrane filter

## Abstract

The aim of the present study was to study the efficiency of different techniques used for nanosizing liposomes. Further, the aim was also to evaluate the effect of process parameters of extrusion techniques used for nanosizing liposomes on the size and size distribution of the resultant liposomes. To compare the efficiency of different nanosizing techniques, the following techniques were used to nanosize the liposomes: extrusion, ultrasonication, freeze-thaw sonication (FTS), sonication and homogenization. The extrusion technique was found to be the most efficient, followed by FTS, ultrasonication, sonication and homogenization. The extruder used in the present study was fabricated using readily available and relatively inexpensive apparatus. Process parameters were varied in extrusion technique to study their effect on the size and size distribution of extruded liposomes. The results obtained indicated that increase in the flow rate of the extrusion process decreased the size of extruded liposomes however the size homogeneity was negatively impacted. Furthermore, the liposome size and distribution was found to decline with decreasing membrane pore size. It was found that by extruding through a filter with a pore size of 0.2 µm and above, the liposomes produced were smaller than the pore size, whereas, when they were extruded through a filter with a pore size of less than 0.2 µm the resultant liposomes were slightly bigger than the nominal pore size. Besides that, increment of extrusion temperature above transition temperature of the pro-liposome had no effect on the size and size distribution of the extruded liposomes. In conclusion, the extrusion technique was reproducible and effective among all the methods evaluated. Furthermore, processing parameters used in extrusion technique would affect the size and size distribution of liposomes. Therefore, the process parameters need to be optimized to obtain a desirable size range and homogeneity, reproducible for various in vivo applications.

## 1. Introduction

One of the key parameters that can influence the in vivo behavior of liposomes is the liposome size. Previous studies have shown that liposomes with diameters >0.1 µm are opsonized more rapidly and at a higher extent than their smaller counterparts and, therefore, are cleared more rapidly by the reticuloendothelial system [1,2]. In addition, the particle size also substantially alters the biodistribution of liposomes [3]. Consequently, in order to prolong the liposome circulation time, several techniques, such as sonication [4], extrusion [5,6] and homogenization [7,8], have been devised to reduce the liposome size. Among these, extrusion method bears several advantages [9]; it yields liposomes with relatively homogeneous size distributions; it is reproducible; the processing is rapid; and it is relatively a gentle process.

Extrusion is a technique where the liposome suspension is passed through a membrane filter of defined pore size [10]. An extruder, a machine equipped with a pump which pushes fluids through the membranes, can be employed to accomplish the extrusion process [11]. Numerous studies on extrusion using various devices have been reported. Examples of devices used include: Lipex extruder [12,13,14,15], Extruder^®^ [16], Avestin Liposo-Fast™-50 [17], Dispex Maximator^®^ [17,18], Nuclepore 24 mm filter holder [10,19], Millipore high pressure filter holder [20], and Hamilton syringes as mini-extruders [21].

Various parameters of the extrusion procedure such as applied pressure, number of cycles and pore size have been found to influence the mean diameter and size distribution (polydispersity) of the liposomes produced [6,9,13,14,18,22]. In the present study, an extruder (different from those previously used) was employed to examine the effect of various factors influencing the size and distribution of extruded liposomes. The assembly basically consisted of a high performance liquid chromatography (HPLC) pump and a refillable guard column equipped with membrane filter. Besides that, a comparison of size and distribution of extruded liposomes with those prepared using other size-reducing methods (sonication, freeze-thaw sonication (FTS) and homogenization) was also performed.

## 2. Materials and Methods

### 2.1. Materials

Pro-liposome (Pro-lipo duo^®^) was obtained from Lucas Meyer, France. Pro-lipo duo^®^ contained 50% unsaturated soybean phosphatidylcholine suspended in hydrophilic medium consisting of glycerol and ethanol.

### 2.2. Instruments

**Figure 1:**
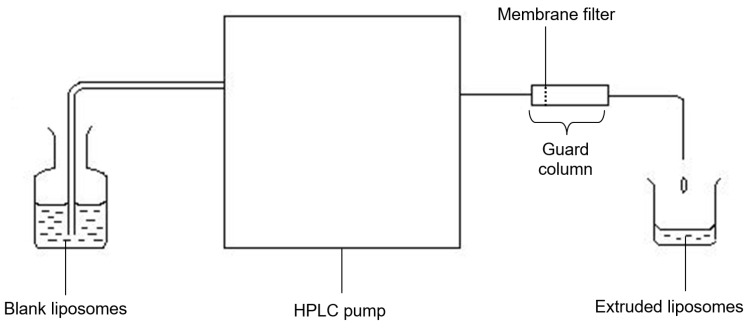
Assembly of instrument used for the extrusion process.

**Figure 2:**
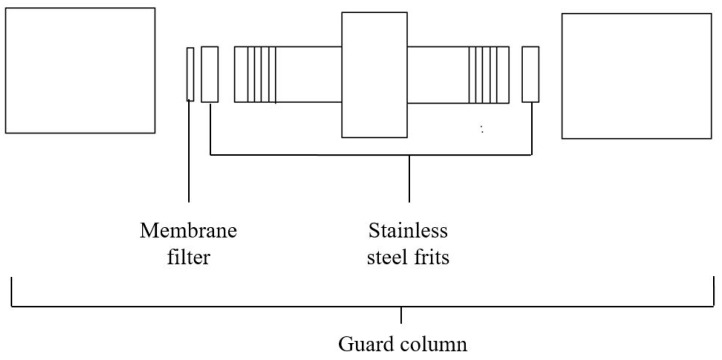
Placement of membrane filter in the guard column.

The extrusion apparatus was made up of a Waters Millipore^®^ 510 HPLC pump (Waters, Milford, MA, USA), a guard column (Upchurch Scientific, Oak Harbour, WA, USA), a stainless steel replaceable frit (filter) with a pore size of 2 µm and polyethersulfone membrane filters of different pore sizes (0.5, 0.2 and 0.1 µm) (Sartorius AG, Goettingen, Germany). The assembly of these apparatus for extrusion process and placement of the membrane filter in the guard column are shown in Figure 1 and Figure 2, respectively.

### 2.3. Preparation of Liposome Suspension

The blank liposome (with no encapsulated drug) was prepared by hydrating the Pro-lipo duo^®^ with purified water from a water purification system (Milli-Q Plus, Millipore, Bedford, MA, USA). The Pro-lipo duo^®^ consisted of 50% unsaturated soybean phosphatidylcholine suspended in hydrophilic medium consisting of glycerol and ethanol. The purified water was used to ensure that the concentration of contaminants in the suspension was negligible. The amount of purified water added was two parts to one part of the pro-liposome. The mixture was then stirred at a moderate speed using a hot plate stirrer (502-P, PMC Industries, Inc., San Diego, CA, USA) for 30 min at room temperature. Prior to use, the suspension of resultant blank liposome was further diluted with five parts of purified water (relative to the pro-liposome) and stirred for another 5 min to produce a homogenous suspension. The lipid concentration in the final dispersion was 6.25% *w/v*.

### 2.4. Extrusion Procedure and Influence of Process Parameters

The liposomal suspension was pushed through the membrane filter five times. Samples eluted during the initial 5 min of each cycle were discarded and aliquots were collected after each cycle for the determination of liposome size and distribution. Several process parameters have been varied to study their influences on the size and distribution of the extruded liposomes. They are summarized as follows:

(a) Flow rate

The liposome suspension was passed through a 0.2 µm membrane filter with the pump operating at different flow rates, namely: 1, 5 and 9 mL/min.

(b) Membrane pore size

The liposome suspension was passed through a membrane filter of different pore sizes, i.e., 2, 0.5, 0.2 and 0.1 µm. The extrusion process was performed with the pump operating at 5 mL/min.

(c) Temperature

The liposome suspension was passed through a membrane filter at varying temperatures of 25, 30, 35, 40, 45 and 50 °C. Water bath was used to maintain the desired temperature of the liposomes. The extrusion process was performed with 0.2 and 0.1 µm membrane filters and a flow rate of 5 mL/min.

All extrusion processes were carried out at ambient room temperature of approximately 25 °C.

### 2.5. Comparison with Size Reducing Methods

The size of the liposome suspension was also reduced using other size-reducing methods such as sonication for 30 min (Sonorex Super, Bandelin Electronic, Berlin, Germany), ultrasonication for 30 min (Digital PRO, Beijing, China), homogenization at 13,500 rpm for 30 min (Ultra-Turrax^®^ T25, Janke & Kunkel GMBH & Co. KG, Staufen im Breisgau, Germany) and FTS for 10 cycles. In an FTS cycle, the liposome suspensions were rapidly frozen using an acetone-dry ice (solid carbon dioxide) bath for 3 min, then thawed rapidly in a water bath (40 °C) for 3 min and lastly, sonicated (Sonorex Super, Bandelin Electronic, Berlin, Germany) for 5 min at room temperature. All the products obtained from these different methods were compared in terms of size, size distribution and physical stability (in terms of particle size changes) over 24 weeks. Those produced by the extrusion method described in Section 2.4 used a 0.1 µm membrane filter. They were compared after they were freshly prepared and at 1, 2, 3, 4, 8, 12 and 24 weeks of storage at 4 °C.

### 2.6. Particle Size Analysis

The liposome size and size distribution were determined by photon correlation spectroscopy (Zetasizer 1000HS, Malvern Instruments Ltd., Malvern, Worcestershire, UK). Prior to measurement, 10 µL of liposome suspensions were dispersed with 500 µL purified water in a low volume disposable sizing cuvette. The particle size and size distribution were measured as ZAve and polydispersity index (PDI) respectively. For each preparation, three measurements were taken on two separate samples. Standard error mean (SEM) of the average was used to estimate the repeatability of the measurements.

### 2.7. Statiscal Analysis

A student *t*-test or one-way ANOVA was used to compare the average particle size where appropriate. A *p* value of <0.05 was considered to be significant.

## 3. Results

### 3.1. Influence of Process Parameters

#### 3.1.1. Flow Rate

**Figure 3:**
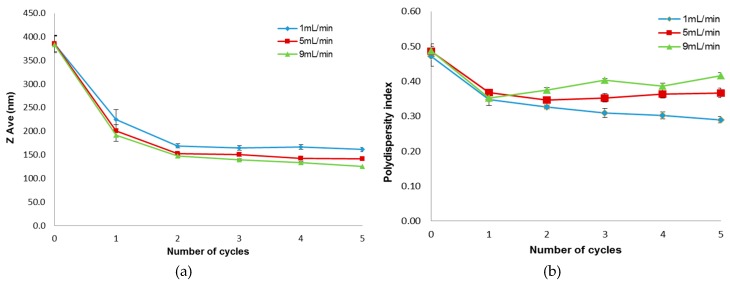
Influence of flow rate on the (**a**) particle size and (**b**) polydispersity index of extruded liposomes (Mean ± SEM, *n* = 6).

The influence of flow rate on the particle size of the extruded liposomes is shown in Figure 3a. It is apparent from the plots that the size of the liposomes was decreased when the number of extrusion cycles was increased. Statistically there was a marked decrease in size after the first and second cycles (*p* < 0.05) and thereafter at cycles 3, 4 and 5 the decrease in size became negligible. Hence, under the conditions studied, the reduction in size of the liposomes from approximately 380 nm to less than 200 nm could be achieved with two extrusion cycles. Further extrusion cycles did not cause any appreciable further decrease in size. The size of extruded liposomes was found to decrease with increasing flow rate of the extrusion process. A total reduction of 12.3% and 22.7% in ZAve values was observed when the flow rate used was increased from 1 mL/min to 5 and 9 mL/min, respectively. The observation of liposomal size reduction with increasing flow rates is consistent with those reported previously by Mason and Bibette [23] as well as Hunter [22]. These studies showed that the size of structures produced decreased with increasing the shear rate, which is most directly related to the flow rate used.

Figure 3b shows the influence of flow rate on the polydispersity index of extruded liposomes. It is apparent from the plots that the relative polydispersity worsened as the flow rate used was increased. Moreover, the number of extrusion cycles did not appear to have any significant effect on the polydispersity index. The increase in the polydispersity values indicates that the homogeneity of liposome size decreases with increasing flow rate.

#### 3.1.2. Membrane Pore Size

**Figure 4:**
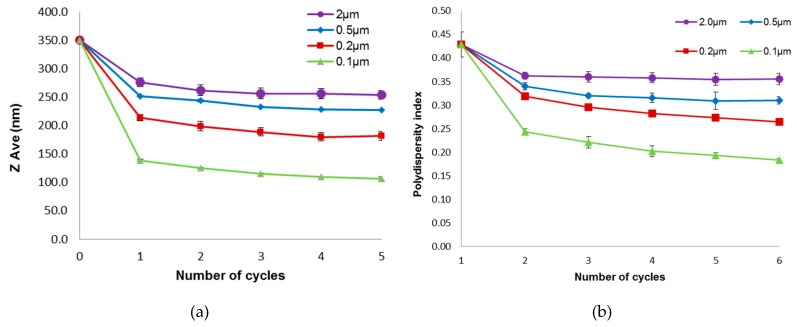
Influence of membrane pore size on the (**a**) particle size and (**b**) polydispersity index of the extruded liposomes (Mean ± SEM, *n* = 6).

The influence of membrane pore size on the particle size of the liposomes is presented in Figure 4a. It is apparent from the plots that decreasing the membrane pore size resulted in a corresponding decrease in the size of the liposomes (*p* < 0.05). Also, the size of the extruded liposomes decreased rapidly during the first two cycles through the membrane (*p* < 0.05) and then leveled off to a relatively constant ZAve value with further extrusion cycles (*p* > 0.05). When relatively big membrane pore sizes (0.2 µm and above) were used, the resulting ZAve values of the liposomes were smaller than the pore size of the membranes. In contrast, a small pore size (0.1 µm) membrane filter resulted in ZAve values that were slightly bigger than the nominal pore size of the membrane. The results obtained in the present study are in accordance with other studies reported in the literature which investigated the relationship between the particle size of liposomes and the pore size of the extruding equipment [9,14,17].

Figure 4b shows the influence of membrane pore size on the polydispersity index which is a measure of the size distribution of the liposomes. From the plots, the polydispersity index of the extruded liposomes was found to be directly related to the membrane filter pore size. Decreasing the filter pore size resulted in a decrease in the polydispersity index of the liposomes. It is also apparent from Figure 4b that the number of extrusion cycles did not have any appreciable effect on the polydispersity index except for the extrusion using 0.1 µm membrane pore size, where the polydispersity index was observed to decrease relatively more than the other pore sizes with each increase in extrusion cycle. In all cases however, a decrease was observed after the first extrusion.

#### 3.1.3. Temperature

**Figure 5:**
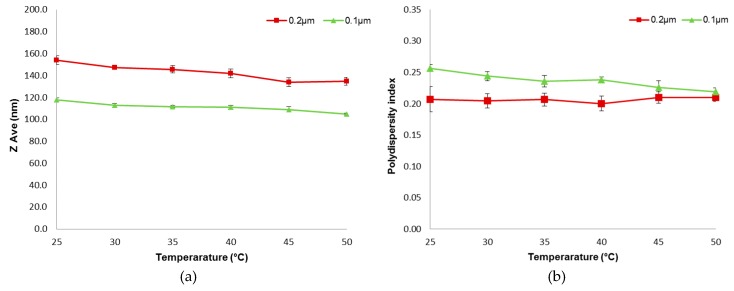
Influence of temperature on the (**a**) particle size and (**b**) polydispersity index of the extruded liposomes (Mean ± SEM, *n* = 6).

Figure 5a shows the influence of temperature on the particle size of the extruded liposomes. It is apparent from the plots that gradually increasing the extrusion temperature only caused very slight decrease in the liposome sizes (*p* < 0.05). Hence, the use of higher temperature does not appear very effective in achieving size reduction of the liposomes using the extrusion technique being investigated. The extrusion temperatures employed in the present study were all above the gel-fluid transition temperature (*T*_c_, which is approximately −15 °C to −30 °C) to ease the extrusion process.

The influence of temperature on the polydispersity index of extruded liposomes is depicted in Figure 5b. The size distribution of the preparations does not seem to be affected by the temperature used. Although there seems to be a decreasing trend in polydispersity in liposomes extruded with membrane pore size of 0.1 µm, the difference was rather small.

### 3.2. Comparison with Other Size Reducing Methods

Table 1 summarizes the size and polydispersity of the blank liposomes before (control) and after treatment with various size reducing methods namely sonication, ultrasonication, homogenization, FTS and extrusion. The liposome sample from the extrusion method that is used for the comparison here was extruded at ambient room temperature for five cycles using a 0.1 µm pore size. Also, all the samples were freshly prepared. Among the various methods used, extrusion was found to be the most efficient in reducing the liposome size. A 67.9% reduction in size was observed when the liposomes were pushed through a 0.1 µm membrane filter. This was followed by FTS, ultrasonication and sonication, which gave a 61.2%, 26.7% and 15.0% reduction in size, respectively. Homogenization was found to be the least effective method in reducing the size of blank liposomes, with only a mere reduction of 13.2%. Statistically there was difference in the ZAve values obtained between extrusion and all other methods used except FTS method. The effectiveness of the methods in reducing the size of liposomes can be summarized as follows:

Homogenization < Sonication < Ultrasonication < FTS < Extrusion

It is also obvious from Table 1 that the extruded liposomes have a relatively smaller polydispersity index compared to liposomes produced by other size reducing methods. This indicates that liposomes produced by extrusion method were most homogenous among all the methods used. Despite the differences observed in the size and size distribution, the polydispersity index of all liposomal preparations after various size reduction treatments was within a narrow size range.

**Figure 6:**
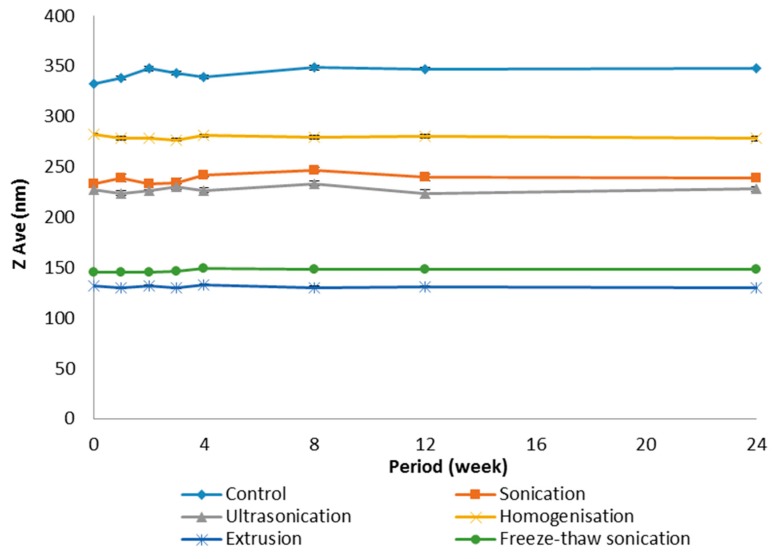
The physical stability of liposomes prepared using the various size reduction treatments kept at 4 °C over 24 weeks (Mean ± SEM, *n* = 6).

The stability profiles of the liposomes kept at 4 °C after size reduction are shown in Figure 6. All the liposomal preparations were found to be stable (in terms of particle size) for at least 24 weeks stored at 4 °C. There was no appreciable change in the particle size of freshly prepared liposomes and those stored for 24 weeks at 4 °C.

## 4. Discussion

Although a variety of techniques can be used for preparing liposomes, typically these methods yield liposomes which are polydisperse, and predominantly greater than one micron in size. Several methods have been proposed to reduce the size and distribution of such liposomal preparations, namely extrusion, homogenization and sonication.

Extrusion is widely used in sizing the liposomes because it is relatively simple and fast to produce liposomes with a homogeneous and controlled average size without any contaminations [14]. Vesicles produced by this technique are extruded under pressure through a filter of selected pore size. Extrusion procedure has advantages over the homogenization and sonication methods, in that the former has a variety of membrane pore sizes made available for producing liposomes in different selected size ranges [10,13,18].

In addition, the size distribution of liposomes can be made quite narrow, particularly by recycling the material through the selected-size filter several times [9]. Nonetheless, U.S. Pat. No. 4, 737, 323 [24] revealed that the membrane extrusion method also has several drawbacks in large-scale processing. According to the patent, there was a tendency for the pores in the membrane to clog, particularly when processing concentrated suspensions and/or when the liposome sizes are substantially greater than the membrane pore sizes. Besides that, the total pore surface area is only about 20% of the total surface area of the membrane, restricting the surface area available for extrusion, and thus limiting the total throughput.

In the present study, the device used for extrusion was easy to use and was capable of generating reproducible results as indicated by the small SEM values. The decrease in size of the liposomes would decrease the drug loading, therefore a balance needs to be obtained between the size reduction required and the drug loading for practical application [2]. Multiple cycles through the extrusion device were shown to increase the size homogeneity of the extruded liposomes [5]. The number of cycles required through the membrane filter depends on the membrane characteristics (pore size, composition and geometry) and the constituents of liposomes [25] and may range from 3 to 10 cycles. It was found that three cycles of extrusion was sufficient to generate homogenous liposomal preparation in the present study, as indicated by the small polydispersity index. In fact, increasing the number of cycles from 3 to 4 and 5 did not cause any further appreciable decrease in both the size and size distribution of the liposomes. Homogenous samples are desirable because the circulation lifetime in the blood of drug-loaded liposomes is size dependent [26].

Generally, the membrane filters come in two types: the conventional “tortuous path” and the “nucleation track” membrane [27]. The “tortuous path” type membrane consists of fibers crisscrossed over each other to give a matrix in which channels are formed from random spaces arising naturally in between the fibers, tracing a tortuous path from one side of the membrane to the other. The average diameter of these channels is controlled by the density of the fibers in the matrix. Due to the convoluted nature of the channels, the filter clogs up easily when liposomes of size larger than the channel diameter are passed through [27]. In contrast, “nucleation track” consists of a thin continuous sheet of polymer (usually polycarbonate) in which straight-sided pore holes of exact diameter have been bored through by a combination of laser and chemical etching. As the pores go straight through from one side to the other, they offer much less resistance to material passing through than the tortuous path membranes do. In the present study, polyethersulfone membrane filters of tortuous type were used for the extrusion of liposomes. Clogging in general depends on variables such as lipid composition, purity and concentration, as well as the pressure and flow rates used [25]. Therefore, in order to avoid pore clogging, higher extrusion flow rate and a more dilute liposome suspension were employed. Besides that, the filters were changed after every five cycles of the liposome suspension.

The feasibility of the extruder for size reduction and the variation of the size of liposomes produced were evaluated by employing various process parameters, namely flow rate, membrane pore size and extrusion temperature. In the course of extrusion, the ZAve of the liposomes produced were found to decrease with increasing flow rates. This observation is in agreement with other studies of complex fluids under shear [23,28]. These studies show that the size of structures produced decrease with increasing shear rate, which is most directly related to the flow rate used in the present study. However, the size distribution of the extruded liposomes did not improve with increasing flow rates. This finding is not in accord with a study by Mason and Bibette [23], whereby their oil-water surfactant systems became more homogenous with increasing shear force. The difference observed could be attributed to the different systems being investigated. In the present study, liposome suspensions were used whereas the system studied by Mason and Bibette [23] was more of an oil in water emulsion.

Furthermore, a decrease in ZAve with decreasing membrane pore sizes was observed. When using relatively big pores (≥0.2 µm), the resulting ZAve of the liposomes were smaller than the pore size, whereas smaller pore size (0.1 µm) resulted in liposomes slightly bigger than the nominal pore size. This can be attributed to elastic deformation of the liposome spheres to ellipsoid shape [29]. These elastically deformed ellipsoid particles pass easier through the pores [10]. Besides that, the size distribution improved with decreasing membrane pore size, as indicated by the decreasing polydispersity index values. The results obtained are comparable to those reported previously [9,17]. According to Frisken et al. [12], the pore size is the only factor that influences the polydispersity of liposome vesicles produced by extrusion.

Previous studies have shown that extrusion employing temperature below *T*_c_ was not feasible [13]. The inability to extrude below *T*_c_ is related to the deformation process that must accompany extrusion through the filter pores [13]. This is because the gel-state systems exhibit significantly greater membrane viscosities than liquid-crystalline systems, as reflected by much-reduced lipid lateral diffusion rates [3]. Thus, the temperatures employed in the present study were all above T_c_. The present findings showed that further increase in the extrusion temperature does not affect both the size and size distribution of the extruded liposomes. This observation is consistent with that made previously by Hunter and Frisken [14].

The actual mechanism by which the large vesicles break up into smaller ones remains unclear. One proposed by Clerc and Thompson [30] was based on the breakup of the cylindrical phospholipid bilayer structures of radius R at the end of the pores into smaller cylindrical structures of length λ, where λ = 2π*R*, which then reform into vesicles. Meanwhile, Gompper and Kroll [31] model the mobility of vesicles in pores as a function of the driving field. The model considers vesicles larger than the pore diameter, which must deform to enter the pore. The deformation was assumed to be governed by bending energy only. Besides that, Bruinsma [32] concentrated on the flow of vesicles inside cylindrical pores and shape deformations which might occur inside the pore. Like Gompper and Kroll (1995), he also assumed that the deformation was governed by bending energy only. In a more recent study, Patty and Frisken [33] suggested that the rupture of vesicles were due to the surface tension that exceeds the rupture tension of the membrane. Based on blowing bubbles through a circular opening, this model predicts that the vesicle radius should be inversely proportional to the square root of applied pressure and that the vesicle size should depend on the size of pores used and the lysis tension of vesicles extruded. Appropriately to the present findings, the liposome size reduction during extrusion is best explained by the latter model. Determination of the lipid content in the samples of extruded liposome is also important even though they have not been evaluated in the present study. It is expected that the lipid content of extruded liposome suspensions would decrease, especially after extrusion through the membranes of very small pore size [6].

Although various methods have been employed, extrusion was found to be the most efficient in reducing the liposome size. Besides that, the liposomes produced by extrusions are relatively more homogenous compared to those produced by other size reducing methods. Homogenization is suitable for large-scale production of liposomes. However, the liposome size distribution produced is typically quite broad and variable, depending on the number of homogenization cycles, pressures, and ionic strength [7]. Also, the processed fluid has a potential to pick up metal and oil contaminants from the homogenizer pump [8]. Moreover, size reduction with homogenization was not as efficient as extrusion method.

On the other hand, sonication, or ultrasonic irradiation is useful especially for preparing SUVs in the 0.025 to 0.08 micron size range. Nonetheless, it may not be amenable to large-scale production, because of a prolonged duration of sonication and the subsequent heat build-up that can lead to peroxidative damage of the lipids and degradation of the solute molecules to be entrapped [34]. On top of that, when a sonication probe is used, high energy sonication can cause probe erosion. Conversely, low energy sonication is slow and it can be destructive to phospholipid molecules [27]. Therefore, sonication is also not the method of choice for size reduction. In FTS method, the freezing and thawing process was aimed at producing physical disruption to the liposomal phospholipid bilayers [27,35] while the sonication process was intended to reduce the size of liposomes [36]. The present findings suggest that the capacity of the FTS method is comparable to that of the extrusion method in reducing the size of liposomes.

Overall, the liposomal preparations were found to be stable (in terms of particle size) for at least 24 weeks stored at 4 °C. There was no appreciable change in the particle size of freshly prepared liposomes and those stored for 24 weeks at 4 °C.

## 5. Conclusions

The device used for extrusion in this study was easy to use and was capable of generating reproducible results as indicated by the small SEM values. Three cycles of extrusion was sufficient to generate homogenous liposomal preparation. The size of extruded liposomes was found to be influenced by the extrusion flow rate and membrane pore size. Increasing the flow rate and reducing the membrane pore size reduced the size of extruded liposomes. Further increase of temperature above *T*_c_ has limited effect on the size of extrusion liposomes. The size distribution or polydispersity was more significantly affected by the membrane pore size.

Among the various size reducing methods attempted, extrusion technique was found to be most effective, giving a 67.9% reduction in liposome size. The extrusion technique also produced the smallest and most homogenous liposomes. Despite the differences observed in the size and distribution, all the liposomal preparations displayed a narrow polydispersity index and were found to be stable for at least 24 weeks stored at 4 °C.

## Figures and Tables

**Table 1:** Particle size of liposomes after treatment with various size reduction methods (Mean ± SEM, *n* = 6).

Size Reduction Method	ZAve (nm)	Polydispersity Index
Control	321.6 ± 12.9	0.4 ± 0.0
Homogenization	279.1 ± 15.8	0.3 ± 0.0
Sonication	273.5 ± 15.3	0.4 ± 0.1
Ultrasonication	235.8 ± 8.1	0.4 ± 0.0
Freeze-thaw sonication (FTS)	124.7 ± 0.7	0.4 ± 0.0
Extrusion	103.3 ± 13.5	0.2 ± 0.2
